# DEMENTIA AND LONG-RUN CHANGES IN FINANCIAL HEALTH AMONG US HOUSEHOLDS

**DOI:** 10.1093/geroni/igae098.1272

**Published:** 2024-12-31

**Authors:** Jing Li, Amy Kelley, Kathleen McGarry, Lauren Nicholas, Jonathan Skinner

**Affiliations:** University of Washington, Seattle, Washington, United States; National Institute on Aging, Baltimore, Maryland, United States; Stony Brook University, Stony Brook, New York, United States; University of Colorado School of Medicine, Aurora, Colorado, United States; Dartmouth College, Hanover, New Hampshire, United States

## Abstract

We use two decades of the U.S. Health and Retirement Study data to compare long-run trajectories in household finances between individuals who eventually developed dementia and those who did not. In prior work, we find large and steady divergence in household wealth that starts 6-8 years prior to dementia onset. In the current paper, we investigate several possible hypotheses for these changes. By examining detailed categories of household income and spending, we find that long-run divergence in wealth between dementia and non-dementia groups are not explained by higher out-of-pocket healthcare expenses or intentional “spend-down” to qualify for Medicare coverage of nursing home residence. Rather, our findings suggest that they are driven by earlier exit from the labor force and potentially suboptimal investment decisions, which negatively affect long-term income in multiple areas among the dementia cohort relative to their counterparts. We also find that these patterns are more prominent among individuals with higher socioeconomic status.

